# Hydrocephalus-Associated Hyponatremia: A Review

**DOI:** 10.7759/cureus.22427

**Published:** 2022-02-21

**Authors:** Chao Li, Iveth Mabry, Yasir R Khan, Michael Balsz, Rodolfo J Hanson, Javed Siddiqi

**Affiliations:** 1 Neurosurgery, Desert Regional Medical Center, Palm Springs, USA; 2 Pharmacy, Arrowhead Regional Medical Center, Colton, USA; 3 School of Medicine, California University of Science and Medicine, Colton, USA; 4 Anesthesiology and Perioperative Medicine, Riverside University Health System Medical Center, Moreno Valley, USA; 5 Neurosurgery, Riverside University Health System Medical Center, Moreno Valley, USA; 6 Neurosurgery, Arrowhead Regional Medical Center, Colton, USA; 7 Neurosurgery, California University of Science and Medicine, Colton, USA

**Keywords:** syndrome of inappropriate adh secretion, adh, normal-pressure hydrocephalus, ventriculoperitoneal shunt, hyponatremia, hydrocephalus

## Abstract

Hydrocephalus is the pathological accumulation of cerebrospinal fluid within the ventricles of the brain. Hydrocephalus may be broadly divided into three categories: congenital, acquired, or other. Hyponatremia, serum sodium level <135 meq/ml, may be caused by dilution (e.g. syndrome of inappropriate antidiuretic hormone (SIADH)), depletion (e.g. cerebral salt wasting (CSW)), or delusion (e.g. psychogenic water intake) etiologies. This review discusses “hydrocephalus-associated hyponatremia” as a clinical entity, distinct from SIADH and CSW.

Some experts believe that in hydrocephalus patients, increased pressure on the hypothalamus leads to the release of antidiuretic hormone (ADH), which in turn causes hyponatremia. The true etiology of hyponatremia is critical to diagnose, as it will determine the treatment. So while both SIADH and CSW may result in hyponatremia, the former is treated with fluid restriction, while the latter requires fluid repletion; treating SIADH as CSW, and vice versa, will exacerbate the hyponatremia.

The etiology and severity of hyponatremia will determine the management. For hydrocephalus-associated hyponatremia, treating the underlying problem (i.e. hydrocephalus) is the mainstay of therapy. Theoretically, treatment of hydrocephalus-related hyponatremia with CSF-diversion procedures should relieve the pressure on the hypothalamus, mitigating ADH production, which in turn will decrease sodium excretion and ameliorate the hyponatremia.

## Introduction and background

Hydrocephalus, hyponatremia, or a combination of these is frequently encountered in neurosurgical patients. However, hydrocephalus-associated hyponatremia is thought to be relatively rare and there is no current consensus of the standard treatment of hydrocephalus-associated hyponatremia. This review article is intended to elucidate the current status and possible direction of study and treatment for hydrocephalus-associated hyponatremia.

Hydrocephalus

Hydrocephalus is defined as the abnormal accumulation of cerebrospinal fluid (CSF) within the ventricles of the brain [1]. The supraoptic nucleus of the hypothalamus reacts to changes in the osmolarity of the blood by secretion of antidiuretic hormone (ADH) [2]. Neurosecretory cells produce and release ADH onto the proximal portion of the hypophyseal portal system within the neurohypophysis. An elevated serum osmolarity drives increased production of ADH within the cells of the supraoptic nucleus. Then ADH is released into the bloodstream and acts at the distal convoluted tubules of renal nephrons, leading to reabsorption of more water and subsequent homeostasis of the serum osmolarity. Several case studies have shown that normal pressure hydrocephalus (NPH) induced the syndrome of inappropriate antidiuretic hormone (SIADH) release, suggesting a possible mechanical pressure related to ADH release as well [3-5].

As often the case with issues of bodily fluid excess, the underlying problem is generally one of overproduction, under secretion, or some combination of the two. Overproduction of CSF is quite rare; however, the major distinction usually becomes one of obstructive versus communicating hydrocephalus [1].

The types of hydrocephalus can be divided into congenital or acquired and those which do not fit precisely into either category (Table 1).

**Table 1:** Types of hydrocephalus with examples. SAH, subarachnoid hemorrhage; IVH, intraventricular hemorrhage; NPH, normal pressure hydrocephalus.

Type of hydrocephalus	Example
Congenital	Chiari I, Chiari II, primary aqueduct stenosis, secondary aqueduct gliosis, Dandy-Walker syndrome, and X-linked inherited disorders.
Acquired	Infectious (post-meningitis and cysticercosis), post-hemorrhagic (post-SAH, post-IVH), secondary to a mass, post-operative, neurosarcoidosis, “constitutional”, and associated with spinal tumors.
Other	NPH, entrapped 4th ventricle, and arrested hydrocephalus.

Hyponatremia

Hyponatremia is defined as a serum concentration of less than 135 meq/mL with the most common causes being SIADH and cerebral salt wasting (CSW) [1,6,7]. Signs and symptoms are dependent on severity and onset of hyponatremia and include, but are not limited to, nausea, vomiting, lethargy, confusion, seizure, brain swelling, and death, and a patient can also be asymptomatic [1,3,6-8]. In SIADH, hyponatremia is due to a decrease or no change in sodium, in addition to an increase or no change in water, therefore, resulting in a relative euvolemic or hypervolemic, hypotonic hyponatremia. There are several causes for SIADH including malignant tumors, CNS disorders, pulmonary disorders, medications, and endocrine disturbance [1,9]. CSW is caused by the excessive excretion of sodium in the urine, therefore, leading to depletional hyponatremia [1,7]. Although both CSW and SIADH cause hyponatremia, they are generally treated in opposing ways. CSW is generally treated via fluid repletion whereas SIADH is generally treated with fluid restriction [1,7]. Additionally, treatment is also dependent on the severity and onset of hyponatremia.

## Review

Connection between hydrocephalus and hyponatremia

ADH, which is also known as arginine vasopressin (AVP), is a hormone produced in the hypothalamus. Release of ADH leads to increased permeability of water through the collecting ducts, resulting in water retention and decreased excretion [1]. As a result, urine is more concentrated and serum sodium is more diluted, resulting in dilutional hyponatremia. Normally, ADH release is triggered by an increase in serum osmolality, intravascular volume depletion, and glucocorticoid deficiency [1]. On the other hand, in patients with hydrocephalus, there is an increase of mechanical pressure against the ventricles due to the buildup of CSF. Consequently, the mechanical pressure on the hypothalamus causes the release of ADH despite plasma osmolality, which then results in hyponatremia due to SIADH [4,5,10].

In a case report by Yoshino et al., a 79-year-old female was diagnosed with idiopathic NPH with hyponatremia caused by SIADH [3]. When this patient was experiencing hyponatremia, high levels of ADH were seen even though the plasma osmolality was low. Furthermore, results of a brain MRI showed some of the ventricles were enlarged, leading to the belief that the hyponatremia may have been due to the mechanical pressure on the hypothalamus caused by the enlargement of the third ventricle [3].

In a case report by Kumar et al., a 70-year-old male had hyponatremia and low serum osmolality [4]. This patient had a brain CT, which showed hydrocephalus. The hyponatremia was believed to be due to SIADH since this patient also had decreased osmolality with inappropriately increased urine osmolality in addition to natriuresis. SIADH in this patient was also believed to be due to the mechanical pressure on the hypothalamus from the third ventricle. Because of this, Kumar et al. concluded that SIADH should be suspected in patients with hyponatremia who also have NPH [4].

In a case report by Olatunbosun, a 78-year-old female was reported with hyponatremia [5]. A brain MRI showed hydrocephalus. Additionally, this patient also presented with other symptoms for NPH. Furthermore, this patient was also found to have low plasma osmolality and low urine osmolality, with a serum ADH level of 6.2 pg/mL [5].

Management of hydrocephalus and hyponatremia

Current Guidelines for the Management of Hydrocephalus

MRI is the first modality to diagnose and confirm the cause and treatment of hydrocephalus, though a CT scan is used in emergency patients [11,12]. Genetic testing and counseling are done for congenital hydrocephalus and CSF analysis is done to rule out residual infection [12].

Surgical intervention remains the most appropriate treatment for hydrocephalus [1,12]. Diuretic use (acetazolamide) for premature infants with hydrocephalus has proven effective as an adjunctive measure [1,12]. Spinal taps can be employed in instances of communicating hydrocephalus. If reabsorption has not resumed when CSF protein concentration is below 100 mg/dL, shunting will likely be necessary [1,12]. Third ventriculostomy is useful for patients with obstructive hydrocephalus [1,12-15]. Shunting of the CSF to other body cavities is a more long-term solution and ventriculoperitoneal (VP) shunts are most common. Ventriculopleural shunts may be implanted in patients in whom the peritoneum is not an option. Ventriculoatrial (VA) shunts are preferred for patients with abdominal and cardiac abnormalities [1,12]. Other terminal locations include the gall bladder, ureter, urinary bladder, and cisternal space [1,12].

For cases of obstructive hydrocephalus, the solution is to open the obstruction. For instances of excessive production of CSF, choroid plexectomy can be considered in rare circumstances such as choroid plexus hyperplasia [12,16].

Current Guidelines for the Management of Hyponatremia

Non-hydrocephalus-associated hyponatremia management: Hyponatremia is treated based on onset, symptoms, and severity of hyponatremia. Acute hyponatremia is characterized as a hyponatremia duration of less than 48 hours, whereas chronic hyponatremia is characterized as a duration greater than 48 hours [8]. Additionally, when correcting hyponatremia, the rate of sodium increase should not exceed 10 mmol/day (10 mEq/day) due to the risk of osmotic demyelination syndrome (ODS) [8]. In specific patient populations, the rate of sodium increase is further limited to 8 mmol/day [8]. The treatment algorithm for hyponatremia can be found in Table 2, which is based on the United States (US) guidelines [8].

**Table 2:** Treatment algorithm for hyponatremia based on US guidelines (2013). ^a^ In liver cirrhosis, restrict to patients where the potential benefit outweighs the risk of worsened liver function [8].

Subject	US guidelines	
Acute or symptomatic hyponatremia	Severe symptoms	Bolus 3% NaCl (100 ml over 10 minutes x 3 as needed)
	Moderate symptoms	Continuous infusion 3% NaCl (0.5-2 ml/kg/hour)
Chronic hyponatremia	Syndrome of inappropriate antidiuretic hormone	1st line: fluid restriction. 2nd line: demeclocycline, urea, or vaptan
	Hypovolemic hyponatremia	Isotonic saline
	Hypervolemic hyponatremia	Fluid restriction, vaptans^a^

Hydrocephalus-associated hyponatremia management: When other medical causes of hyponatremia are ruled out, hyponatremia can improve with treatment for hydrocephalus. One unique aspect of hydrocephalus-associated hyponatremia is the central role that SIADH plays. It has been theorized that hydrocephalus, through the expansion of the third ventricle, may be applying mechanical pressure to the supraoptic and paraventricular nuclei of the hypothalamus [5]. These nuclei are responsible for the production of ADH and it is believed that the mechanical pressure causes an uncontrolled release of the hormone causing serum hypoosmolality and serum hyponatremia. These relationships mean that managing one condition has ramifications beyond that one condition. The resolution of hydrocephalus, therefore, should relieve the pressure on the hypothalamus, which then should reduce the release of ADH leading to resolution of serum hypoosmolality and serum hyponatremia.

Yoshino et al. reported that the placement of a VP shunt led to the resolution of the patient’s hyponatremia [3]. If further research bears out the same result, then management of hyponatremia will be with reduction of intracranial pressure.

A rare case of neonatal hyponatremia with progressive hydrocephalus was reported [17]. It is recommended that neonatal hyponatremia with progressive hydrocephalus should be treated with early neurosurgical intervention if other etiologies of electrolyte disturbance cannot be identified.

Although there are patients who are responsive to VP shunting, others may worsen or do not want to have surgery. In such cases, oral antihypertensive medications may be beneficial in NPH due to the reduction of cerebral systolic arterial blood pressure and increased diastolic blood flow, which may result in protection against pressure on the periventricular tissues [18]. Additionally, to manage the SIADH, a fluid restriction may be utilized as it has been shown to improve SIADH in this setting [3,19].

Sometimes the patient presents with hydrocephalus-associated hyponatremia simultaneously. Nevertheless, these may simply be due to different etiology. It is critical to identify them and treat them accordingly. A rare case of a primary CNS lymphoma presented with both hydrocephalus and hyponatremia [10]. In this case, hydrocephalus was due to infiltration of CNS lymphoma to ventricles, whereas the lymphomatous involvement of the pituitary stalk may cause cortisol deficiency and hypothyroidism with symptomatic hydrocephalus. In this case, surgery is not indicated, whereas chemotherapy of CNS lymphoma addressed both hydrocephalus and hyponatremia. Similarly, tuberculous meningitis patients may present with similar symptoms of hydrocephalus and hyponatremia, which need non-surgical intervention [20].

A study done by Chou et al. showed that hyponatremia was found in 22.6% of patients with NPH [21]. If the results of this paper are confirmed by other researchers, it points to a need to make detection and management of hyponatremia a standard part of the care of patients with NPH. This paper confidently proclaimed that hyponatremia in NPH is “not uncommon,” and this should prompt more research on how common hyponatremia is among NPH patients and hydrocephalus in general.

Prognosis and outcomes

Primary neurological and neurosurgical conditions that are accompanied by hyponatremia tend to have poor outcomes across the age spectrum [22]. Hyponatremia adds to the complexity of conditions that face patients and adds a major stressor to patients’ homeostasis. Hyponatremia is often seen in about 50% of neurosurgical patients, which increases the risk for complications like vasospasms, cerebral edema, altered mental status, and convulsions to occur [23-25]. Therefore, having hyponatremia complicates hydrocephalus leading to poorer outcomes and increased complications.

## Conclusions

Both hydrocephalus and hyponatremia are commonly encountered in neurosurgical/neuro intensive care environments. They are both well studied with well-established treatment guidelines including medical and surgical interventions. Several journal articles have reported hydrocephalus-associated hyponatremia and several different theories have been proposed for this mechanism. The most prominent one is that hydrocephalus results in pressure on the hypothalamus nuclei, which triggers the release of ADH, and subsequent hyponatremia. This theory is based on the radiographic finding of the dilated ventricle with assumed pressure on the hypothalamus, and resolution of hyponatremia after surgical decompression of the hydrocephalus. Though there appears to be anecdotal evidence in favor of the idea that hydrocephalus-induced hypothalamic release of ADH is real, as of now, there is no animal model to prove the accuracy of this hypothesis.

Based on these various findings regarding hydrocephalus-related hyponatremia, it is difficult to generate a standardized workup approach and treatment plan for hydrocephalus-associated hyponatremia. Thus, further study is warranted for further delineation of this relatively rare pathology to further facilitate the guidelines for treatment. As for now, it is recommended that when both hydrocephalus and hyponatremia are present on the same patient, individualized workup and study are indicated. As previously reported, after etiology is identified, the treatment can be drastically different, surgical versus medical, sometimes both. At the minimum, all patients with an unclear etiology of hyponatremia should have brain imaging to rule out hydrocephalus as a possible mechanism of the problem.
